# German Genius and Methods: Is Their Reputation Justified by Their Real Worth?

**Published:** 1917-09-22

**Authors:** 


### GERMAN GENIUS AND METHODS.

### Is their Reputation Justified by their Real Worth?

"Not one single discovery of the first importance in the science or in the art of surgery can be placed to the credit of Germany." Such is the conclusion maintained by Sir Berkeley Moynihan in an article in the *British Medical Journal* (August 11), and supported by an historical record of very high interest. The writer, however, allows that public and even professional opinion in most, if not in all, the countries of the Continent leans in entirely the opposite direction, and would correctly be expressed in the remark of a distinguished Scandinavian surgeon—namely, "All surgical advance in the last fifty years has come from Germany." Sir Berkeley Moynihan has had the opportunity of debating the issue with his Scandinavian *confrère*, and his article is presumably a record of the facts and arguments he was able to advance. The record is certainly a very impressive one, and the manner of its display is distinguished by much fulness of knowledge and great literary skill.

### A Question of Importance.

All the same, the difference between the two opposed propositions has to be explained. If the sentence quoted at the beginning of this article is a true statement of the position, how does it happen that there is so widespread an impression of the value and originality of the German surgical work? Why, in other words, does the general verdict award to Germany a surgical reputation which is wholly undeserved? The answer proposed is of interest in itself, and from it may perhaps be gathered some practical worldly wisdom worthy of being noted for future guidance. It has even a wider range, for it well may be that certain German reputations other than surgical ones are equally without solid foundations, and that they, too, owe their growth to the same ingenious methods which have placed German surgery in a position to which it has no real title.

### The Fruit of Industry.

After all, there must be some reason for an impression which is admittedly widely spread through the civilised world. *Securus judicat orbis terrarum* is a writ the efficacy of which is by no means limited to the world of theology. And in the present instance it must freely be allowed that there is for the prevailing view a basis of real, hard, and enduring work. Only the work, valuable of its kind, has not the qualities which justify the judgment widely passed upon it. The new idea and the original inspiration take origin not in the German mind but elsewhere. But they are given "eager hospitality" in Germany, and are analysed and discussed with untiring industry. Modification of method and alterations of technical procedure are developed and emphasised, and gradually the discovery is given a German dress and appears before the world as a wholly German discovery. German writers—by no means always in bad faith—take this position for granted, and while their writings are industriously crowded with references to the work of their fellow-countryman, the work of others is largely ignored. Tabulation and registration are features of compilations of this order, and they are carried out in a most thorough and elaborate fashion. The impression they produce on those who consult them is considerable, and undoubtedly they are the fruit of great industry and are worthy of recognition.

### Compilation *v.* Discovery.

In this particular respect the German method takes a very high place, and its expansion over large areas keeps the German name prominent in many discussions; and not everyone pauses to distinguish between the industry of the compiler and the inspiration and vision of the genius. The German mark is placed on the whole of the enterprise, and is widely accepted as a guide to the origin of the whole undertaking. No one, as a rule, troubles to explain the true position or to challenge the claim, or, if such a step is taken, it is not sustained by any widespread propaganda. The people who really know are few in number, and the best of them have something to do in the world other than cultivating discussions on priority of discovery. Thus the German claim is often allowed to pass without protest, and the industry which is spent in elaboration and dissection receives the credit which is due to the idea on which these virtues are practised.

### Pretentious Presentation.

Another feature which characterises German scientific literature and which gains for it a reputation in excess of its value is the elaborate and extended discussion which it gives to any subject of which it treats. Not only does it penetrate into every nook and corner to seek out every atom of information which bears on the discussion, but it pretends to a finished and systematic completeness that is, at least not very infrequently, independent of facts. Books are thus produced which both by their bulk and their form appear to be the most full and the most authoritative treatises on the subjects with which they deal. The appeal of these to the young and ambitious student is a very strong one, and if a further experience teaches him that the classifications of his mentor are hard to accommodate with his individual experience, modesty probably prompts him to doubt the latter rather than admit a loss of confidence in an author whose manner is so truly magistral.

### Unknown in Germany.

Sir Berkeley Moynihan illustrates this position by a reference to the numerous and voluminous

German treatises on diseases of the stomach. They are indeed impressive both in their size and in the elaborate and confident classifications they display. But whether these classifications and sub-classifications can be reconciled with clinical facts is quite another matter. Personally we accept Sir Berkeley Moynihan's conclusion that though German writers appear never to have heard the name of Brinton "it is probable that in his small book there is more of the truth of the matter of gastric diseases than in all the interminable treatises published by all the German physicians since his death." A formal detailed and systematic presentation of a subject is much to be desired, provided the facts justify such a display. But even when this is not the case the display makes a great impression upon the unwary and on the modest and eager seeker after truth.

How the German Author is Advertised.

A more mechanical explanation of the spread of German fame is found in the steps which are taken to make German writings widely known. Any German publisher, it seems, will send books and journals on approval to any reputable professional applicant, whereas English or American books can only be obtained on the Continent by purchase outright. A similar spirit is exhibited in the distribution of books for review, with the result that the German author is much more widely advertised in foreign countries than are the writers of other nationalities. Thus to an extent out of proportion to its real value German literature takes a leading place in the spread of knowledge, and gets recognition as the teacher of nations other than the one in which it takes origin. Add to all these influences a repeated and arrogant claim to intellectual superiority, and a tacit allowance of this claim by many who are either too busy or too scornful to challenge it, and we have an explanation of the widespread reputation which Germany undoubtedly possesses as a leader in scientific literature and enterprise. If we dismiss this superiority, as we are well entitled to do, we may perhaps with advantage recognise the qualities of industry and thoroughness which give to it an appearance of validity, and may be less content to cultivate a modest if splendid isolation. Recent events are likely to protect us from another habit to which hitherto we have been accustomed, and in the future "made in Germany" is little likely to be accepted in the English-speaking world as a guarantee of truthfulness, excellence, and culture.

